# Comorbidities and neurosurgical interventions in a cohort with connective tissue disorders

**DOI:** 10.3389/fneur.2024.1484504

**Published:** 2025-01-22

**Authors:** Ilene S. Ruhoy, Paolo A. Bolognese, Jared S. Rosenblum, Randall A. Dass, Navdeep S. Nayyer, Jeffrey D. Wood, John B. Biggins

**Affiliations:** ^1^Division of Neurology, Chiari EDS Center of Mount Sinai South Nassau, Oceanside, NY, United States; ^2^Division of Neurosurgery, Chiari EDS Center of Mount Sinai South Nassau, Oceanside, NY, United States; ^3^Department of Rehabilitation and Human Performance, Icahn School of Medicine at Mount Sinai, New York, NY, United States

**Keywords:** Ehlers-Danlos, Chiari, mast cell activation disorder, craniocervical instability, tethered cord, connective tissue disorders, dysautonomia

## Abstract

**Background:**

Connective tissue disorders (CTDs) are a heterogeneous group of disorders often presenting with a variety of comorbidities including musculoskeletal, autonomic, and immune dysfunction. Some CTDs such as hypermobile Ehlers-Danlos syndrome (hEDS), which is one of the most common, have been associated with neurological disorders requiring surgical intervention. The frequency of these comorbidities in these populations and their subsequent requirement for neurosurgical intervention remains unclear.

**Methods:**

Based on our initial experience with this population, we investigated the presentation rates of specific comorbidities and neurosurgical interventions in a cohort of individuals referred to our institution for evaluation and neurosurgical management of issues secondary to diagnosed or suspected CTDs from 2014 to 2023. Primary diagnoses were made by referring physicians or institutions based on clinical presentation and standard-of-care criteria. We evaluated relationships between diagnoses and surgical interventions by multivariate correlation and intersection plots using the UpSetR package.

**Results:**

Of 759 individuals, we excluded 42 based on incomplete data. From the remaining (total cohort, *N* = 717), 460 (64%) individuals were diagnosed with hEDS, 7 were diagnosed with a CTD other than hEDS, and 250 lacked a formal CTD diagnosis. We found that individuals with hEDS had a higher frequency of certain comorbidities, such as Mast Cell Activation Disorder and Postural Orthostatic Tachycardia Syndrome, and neurosurgical intervention compared to individuals without a CTD diagnosis (unaffected). Of the total cohort, 426 (59%) were diagnosed with Chiari I Malformation, which shared a significant overlap with hEDS. Of those who elected to undergo surgery (*n* = 612), 61% required craniocervical fusion (CCF). Notably, of the 460 individuals diagnosed with hEDS, 404 chose surgical intervention, of which, 73% required CCF for craniocervical instability.

**Conclusion:**

In this retrospective study of individuals referred to our institution for evaluation of CTDs potentially requiring neurosurgical intervention, we defined the frequency of presentation of specific comorbidities that we commonly encountered and revealed the rate at which they required neurosurgical intervention.

## Introduction

Connective tissue disorders (CTDs) are a heterogeneous group of conditions often presenting with a variety of comorbidities (1–3). CTDs, which are linked by connective tissue dysfunction, may have widely variable clinical presentation and arise from a variety of etiologies, including heritable or sporadic genetic variants; however, the exact cause in many of these disorders remains unknown (4–6). For example, Ehlers-Danlos Syndrome (EDS), which is one of the most common CTDs, is further subdivided into 13 variants with distinct clinical presentations and etiologies (7–9). The criteria of the subdivisions of EDS are continuously being refined (8, 10, 11), with hypermobile EDS (hEDS) presenting as the most common variant (approx. 80–90% of all EDS diagnoses) (12, 13). Recent estimates mark the prevalence of hEDS as high as 1-in-500 (14), indicating a more widely affected population than commonly believed. While the majority of EDS subtypes can be linked to a specific genetic variant, hEDS is unique in that it lacks a specific diagnostic genetic marker despite a strong familial association (11, 12, 15). As such, hEDS, a syndrome of multiple coincident pathologies appearing to be centered around connective tissue dysfunction, is established by a comprehensive clinical diagnosis focusing around one primary factor: ligamentous laxity (16).

The association of hEDS with neurological, immunological, and musculoskeletal dysfunction is increasingly being appreciated (8, 16–18). Notably, individuals with symptoms of hEDS often present with concomitant autonomic and immune dysfunctions such as postural orthostatic tachycardia syndrome (POTS) and mast cell activation disorder (MCAD), respectively, whose associations with connective tissue and joint fragility are well documented (10, 19). However, the list of comorbid diagnoses that is often present in individuals with hEDS is not fully appreciated. For example, while it has been noted that hEDS may present with a variety of neurosurgical comorbidities, including Chiari I malformation (CMI) (8, 16, 17, 20, 21), tethered cord syndrome (TCS) (22–26), and styloid hypertrophy (SH) (27, 28), the frequency of these comorbidities in the larger population of individuals with hEDS, their relationship to hEDS, and the need for neurosurgical management remains unclear.

Herein, we investigated the presentation rates of comorbidities and neurosurgical interventions in a cohort of individuals with CTDs who were referred to our institution for evaluation and management.

## Methods

### Patient selection

Individuals with a formal or suspected diagnosis of any CTD were referred to our center for evaluation and management of suspected neurosurgical issues from September 2014 to April 2023. Diagnoses were made by referring physicians or institutions based on clinical presentation and standard-of-care criteria. We included all individuals referred to us with CTDs. For individuals referred with an outside diagnosis of hEDS, we used the Beighton Scoring System, which assesses joint hypermobility on a 9-point scale, to verify the diagnosis; a score of 5 or higher was considered diagnostic (29, 30). Individuals without a formal CTD diagnosis were considered unaffected for the purpose of this study. Individuals were selected for surgical intervention if their score on the Karnofsky Performance Scale (31) related to their chief complaint was 70 or lower at the time of initial evaluation. We excluded individuals with incomplete medical records.

### Retrospective review

We collected individual demographics, diagnoses, and neurosurgical interventions commonly seen in our population. We stratified individuals based on their CTD diagnosis, which included: hEDS, Marfan syndrome, Sjögren’s syndrome, Stickler syndrome, Systemic Lupus Erythematosus, and all hypermobility spectrum diagnoses. Based on our initial experience with this population, we evaluated the presentation rates of 13 diagnoses: CMI, TCS, POTS, MCAD, dysautonomia, myalgic encephalomyelitis/chronic fatigue syndrome (ME/CFS), SH, gastroesophageal reflux disease (GERD), hypothyroidism, gastroparesis, small fiber polyneuropathy (SFPN), post-treatment Lyme disease syndrome (PTLDS), and median arcuate ligament syndrome (MALS). Similarly, based on our experience with this population, we evaluated the following neurosurgical interventions: craniocervical fusion (CCF), posterior fossa decompression (PFD), tethered cord release (TCR), ventriculoperitoneal shunt (VPS), CSF leak repair, styloidectomy, anterior cervical discectomy and fusion (ACDF), and transoral odontoidectomy (TOO). Records were supplemented with patient survey data regarding specific diagnoses and surgeries within their medical history.

### Data analysis

We determined statistical significance among the relationships of the individual diagnoses and surgeries by multivariate correlation analysis (Pearson). Fisher’s exact test and odds ratios with 95% confidence intervals were established when comparing hEDS- and non-CTD-presenting populations. *p*-values <0.05 indicated significance. Analysis was performed through the GraphPad Prism statistical and graphing platform and Microsoft Excel. UpSet plots, which illustrate co-occurrence or mutual exclusion of up to 40 data sets, were generated with the R-programming package UpSetR (32, 33).

## Results

### Demographics

We evaluated 759 individuals referred to our center for surgical management of CTDs. We excluded 42 individuals with incomplete data in the medical record, resulting in a final cohort of 717. Sex demographics skewed approximately 87:13 female-to-male (6.5:1). The average age was 37 years (*SD =* 14; range: 4–74 years) at their first procedure at our institution. Supplementary Table S1 summarizes demographics, diagnoses, and surgeries are for the population included in the study.

### Diagnoses and comorbidities

Retrospective chart review revealed the frequencies of the comorbid conditions that we commonly encounter based on our experience in our population of individuals with CTDs (Table 1). Of note, 460 (64%) were diagnosed with hEDS and 426 (59%) were diagnosed with CMI. In the total cohort, 89% of individuals had a diagnosis of hEDS and/or CMI (or both). Additional frequencies of diagnoses encountered are as follows: TCS (42%), POTS (41%), MCAD (34%), dysautonomia (27%), ME/CFS (22%), SH (20%), GERD (16%), hypothyroidism (14%), gastroparesis (12%), SFPN (11%), PTLDS (7%), and MALS (5%).

**Table 1:** Diagnoses of total cohort and subgroups of individuals presenting with hEDS, lacking hEDS or other CTDs (Unaffected), lacking hEDS but with any additional CTD (Any CTD; EDS neg.), presenting with CMI, and presenting with both hEDS and CMI diagnoses (EDS and CMI).

Diagnosis	Total cohort	hEDS	Unaffected	Any CTD (EDS neg.)	CMI	EDS and CMI
Individuals, *n* (%)	717	460	250	7	426	249
Ehlers-Danlos syndrome	460 (64.2%)	460 (100%)	0 (0.0%)	0 (0.0%)	249 (58.5%)	236 (100%)
Unaffected	250 (34.9%)	0 (0.0%)	250 (100%)	0 (0.0%)	172 (40.4%)	0 (0.0%)
Any CTD (EDS negative)	7 (1.0%)	0 (0.0%)	0 (0.0%)	7 (100%)	5 (1.2%)	0 (0.0%)
Any non-EDS CTD	44 (6.1%)	37 (8.0%)	0 (0.0%)	7 (100%)	21 (4.9%)	16 (6.4%)
Chiari I malformation	426 (59.4%)	249 (54.1%)	172 (68.8%)	5 (71.4%)	426 (100%)	236 (100%)
Tethered cord syndrome	302 (42.1%)	227 (49.3%)	75 (30.0%)	0 (0.0%)	173 (40.6%)	124 (52.5%)
Postural orthostatic tachycardia syndrome	291 (40.6%)	239 (52%)	51 (20.4%)	1 (14.3%)	123 (28.9%)	98 (41.5%)
Mast cell activation disorder	243 (33.9%)	203 (44.1%)	39 (15.6%)	1 (14.3%)	91 (21.4%)	78 (33.1%)
Dysautonomia	191 (26.6%)	166 (36.1%)	24 (9.6%)	1 (14.3%)	92 (21.6%)	81 (34.3%)
Myalgic encephalomyelitis/chronic fatigue syndrome	158 (22.0%)	113 (24.6%)	44 (17.6%)	1 (14.3%)	49 (11.5%)	38 (16.1%)
Styloid hypertrophy	145 (20.2%)	110 (23.9%)	33 (13.2%)	2 (28.6%)	88 (20.7%)	64 (27.1%)
Gastroesophageal reflux disease	116 (16.2%)	92 (20.0%)	23 (9.2%)	1 (14.3%)	73 (17.1%)	54 (22.9%)
Hypothyroidism	97 (13.5%)	72 (15.7%)	24 (9.6%)	1 (14.3%)	52 (12.2%)	38 (16.1%)
Gastroparesis	85 (11.9%)	76 (16.5%)	9 (3.6%)	0 (0.0%)	41 (9.6%)	36 (15.3%)
Small fiber polyneuropathy	77 (10.7%)	65 (14.1%)	12 (4.8%)	0 (0.0%)	40 (9.4%)	34 (14.4%)
Post-treatment Lyme disease syndrome	47 (6.6%)	33 (7.2%)	14 (5.6%)	0 (0.0%)	11 (2.6%)	6 (2.5%)
Median arcuate ligament syndrome	32 (4.5%)	28 (6.1%)	4 (1.6%)	0 (0.0%)	15 (3.5%)	12 (5.1%)

Percentage is based on the individual subgroup population, *n*.

**Figure 1:**
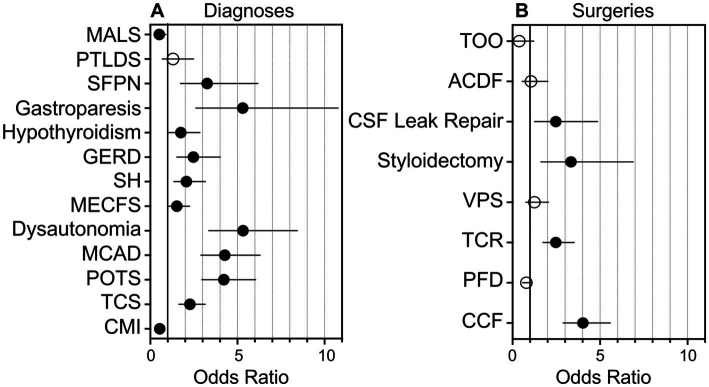
**Graphical representation of the likelihood of individuals with hEDS presenting with comorbid diagnoses or requiring surgical intervention as compared to unaffected individuals**. Graphical representation of the likelihood of individuals with hEDS presenting with comorbid diagnoses or requiring surgical intervention, as determined by odds ratios as compared to unaffected individuals, is shown. **(A)** Odds ratios for individuals with hEDS presenting with diagnoses including Chiari I malformation (CMI), tethered cord syndrome (TCS), postural orthostatic tachycardia syndrome (POTS), mast cell activation disorder (MCAD), dysautonomia, myalgic encephalomyelitis/chronic fatigue syndrome (MECFS), styloid hypertrophy, gastroesophageal reflux disease (GERD), hypothyroidism, gastroparesis, small fiber polyneuropathy (SFPN), post-treatment Lyme disease syndrome (PTLDS), and median arcuate ligament syndrome (MALS) are shown on a grouped plot. **(B)** Odds ratios for individuals with hEDS requiring surgical intervention including craniocervical fusion (CCF), posterior fossa decompression (PFD), tethered cord release (TCR), ventriculoperitoneal shunt (VPS), styloidectomy, CSF leak repair; anterior cervical discectomy and fusion (ACDF), and transoral odontoidectomy (TOO) are shown. Filled circles, *p* < 0.05; open circles: *p* > 0.05; line, 95% confidence interval.

Of the 257 individuals not presenting hEDS, 7 individuals were diagnosed with another CTD. While the remaining 250 individuals were referred to our institution with a suspicion of an underlying CTD, they did not carry a formal diagnosis. Thus, in this study, we categorized these individuals as unaffected. Individuals with hEDS were significantly more likely to present with dysautonomia (*p <* 0.001; OR 5.32, 95% CI: 3.35–8.44) gastroparesis (*p <* 0.001; OR 5.30, 95% CI: 2.61–10.77), MCAD (*p <* 0.001; OR 4.27, 95% CI: 2.90–6.30), POTS (*p <* 0.001; OR 4.22, 95% CI: 2.95–6.04), SFPN (*p <* 0.001; OR 3.26, 95% CI: 1.73–6.17), GERD (*p <* 0.001; OR 2.47, 95% CI: 1.52–4.01), TCS (*p <* 0.001; OR 2.27, 95% CI: 1.64–3.15), SH (*p <* 0.001; OR 2.07, 95% CI: 1.35–3.16), hypothyroidism (*p <* 0.05; OR 1.75, 95% CI: 1.07–2.85), ME/CFS (*p <* 0.05; OR 1.52, 95% CI: 1.03–2.25), and PTLDS (*p >* 0.05; OR 1.30, 95% CI: 0.68–2.48) (Figure 1A). PTLDS had no significant association to either population. The unaffected group was more likely to present with CMI (*p <* 0.001; OR 0.54, 95% CI: 0.39–0.74) and MALS (*p <* 0.05; OR 0.51, 95% CI: 0.30–0.89) than the hEDS group, which may reflect the presence of other undiagnosed congenital syndromes in the unaffected population.

### Intersections of diagnoses

**Figure 2:**
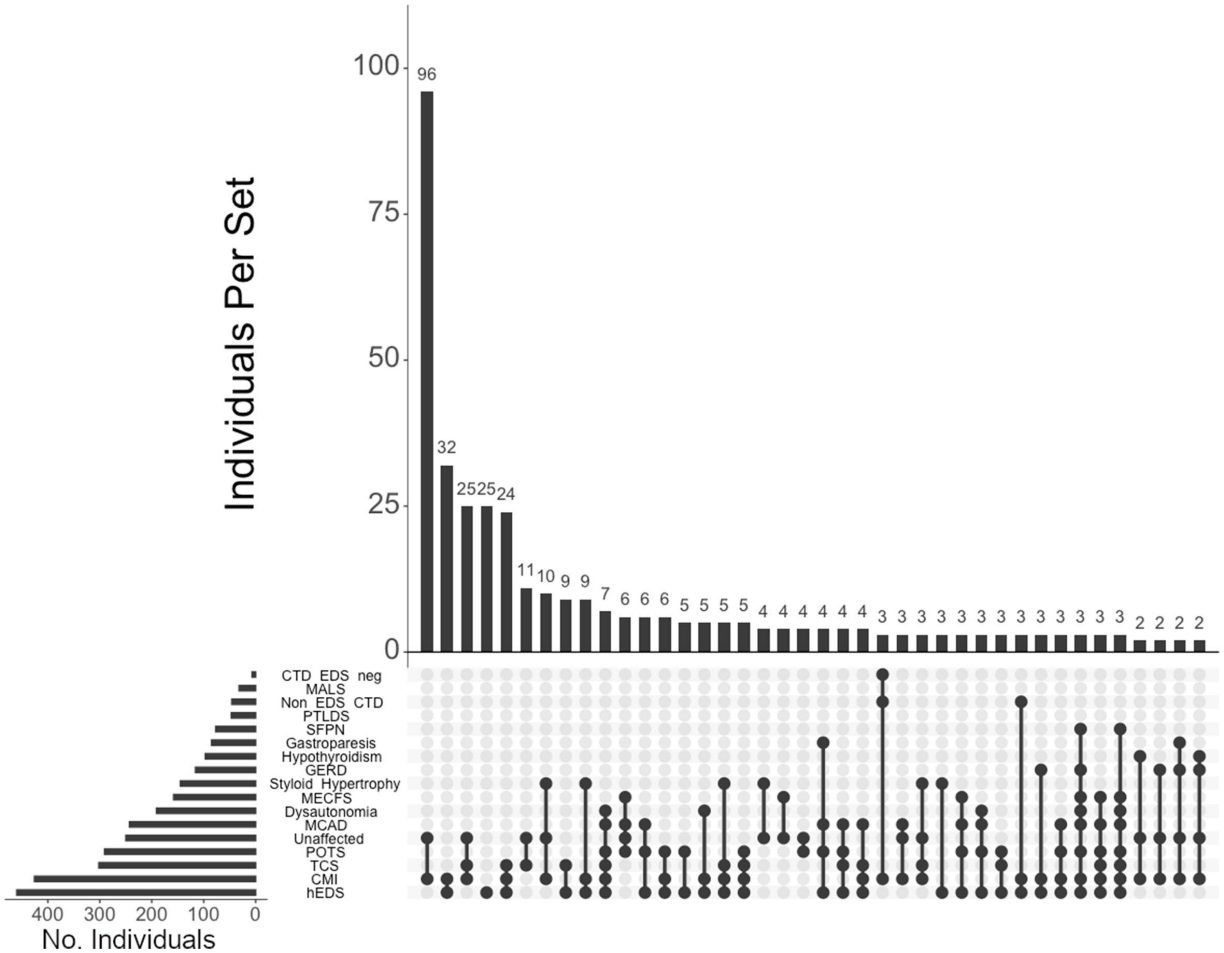
**UpSet plot of diagnoses and comorbidity intersections of the total cohort**. Each column represents the intersection (or lack thereof) of one or more diagnoses, with the number of individuals in each grouping listed on top of each bar. Each row indicates groupings of data (sets) corresponding to the diagnosis listed on the left. Dark circles indicate the placement of a diagnosis within a group. Groupings comprised of two or more comorbidities are indicated by circles connected by a line. The bar graph on the bottom-left indicates the number of individuals specific to that diagnosis, corresponding to the frequency detailed in Table 1. The top 40 intersections are illustrated. Diagnoses: Hypermobile Ehlers-Danlos syndrome (hEDS); Chiari I malformation (CMI); tethered cord syndrome (TCS); postural orthostatic tachycardia syndrome (POTS); those lacking a formal CTD diagnosis (Unaffected), mast cell activation disorder (MCAD); dysautonomia; myalgic encephalomyelitis/chronic fatigue syndrome (MECFS); styloid hypertrophy, gastroesophageal reflux disease (GERD), hypothyroidism, gastroparesis, small fiber polyneuropathy (SFPN), post-treatment Lyme disease syndrome (PTLDS), those presenting CTDs other than hEDS (Non-EDS CTD), median arcuate ligament syndrome (MALS), and those without hEDS who present for another CTD (CTD EDS-neg.).

We visualized intersections of diagnoses using UpSetR, a statistical package for illustrating intersections of multiple data sets (32, 33), and found that the top 5 patterns of intersecting diagnoses among individuals in our cohort were, in decreasing order: (1) CMI in individuals unaffected by CTD (96 individuals, or 13% of the total cohort), (2) CMI in individuals with hEDS (32, or 4%), (3) CMI and TCS in unaffected individuals (25, or 3%), (4) individuals with hEDS (25, or 3%), and (5) CMI and TCS in individuals with hEDS (24, or 3%) (Figure 2).

### Surgeries

**Figure 3:**
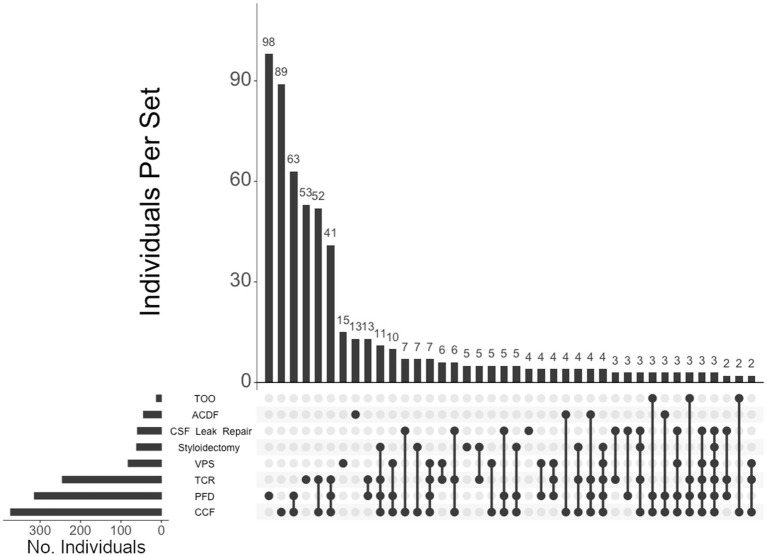
**UpSet plot of the most frequent surgery intersections**. Each column represents the intersection (or lack thereof) of one or more surgeries, with the number of individuals in each grouping listed on top of each bar. Each row indicates groupings of data (sets) corresponding to the surgeries listed on the left. Dark circles indicate the placement of a surgery within a group. Groupings comprised of two or more surgical procedures are indicated by circles connected by a line. The bar graph indicates the number of individuals specific to each procedure, corresponding to the frequency detailed in Table 2. The top 40 intersections are illustrated. Surgical procedures: craniocervical fusion (CCF); posterior fossa decompression (PFD); tethered cord release (TCR); ventriculoperitoneal shunt (VPS); styloidectomy; CSF leak repair; anterior cervical discectomy and fusion (ACDF); transoral odontoidectomy (TOO).

Of the total cohort, 612 (85%) individuals elected to undergo surgery. Neurosurgical interventions that individuals in our cohort underwent are listed in Table 2. Of note, approximately half of the total cohort required CCF surgery (52%), followed by PFD (44%), TCR (34%), VPS (11%), styloidectomy (9%), CSF leak repair (8%), ACDF (6%), and TOO (2%). UpSet plots of intersections indicated that individuals in our cohort most frequently underwent (1) PFD alone (98 patents, or 16% of individuals undergoing surgery), (2) CCF alone (89, or 15%), (3) both CCF and PFD (63, or 10%), (4) TCR alone (53, or 9%), (5) both CCF and TCR (52, or 9%), and (6) CCF, PFD, and TCR (41, or 7%) (Figure 3). Compared to unaffected individuals, those with hEDS had a higher rates of CCF (*p <* 0.001; OR 4.02, 95% CI: 2.89–5.59), styloidectomy (*p <* 0.001; OR 3.34, 95% CI: 1.62–6.90), TCR (*p <* 0.001; OR 2.47, 95% CI: 1.74–3.52), CSF leak repair (*p <* 0.01; OR 2.47, 95% CI: 1.26–4.86), VPS (*p >* 0.05; OR 1.25, 95% CI: 0.76–2.05), and ACDF (*p >* 0.05; OR 1.05, 95% CI: 0.55–2.01), while the unaffected had higher rates of PFD (*p >* 0.05; OR 0.79, 95% CI: 0.58–1.08) and TOO (*p >* 0.05; OR 0.38, 95% CI: 0.12–1.21) (Figure 1B). There were no significant associations between the two subgroups for PFD, VPS, ACDF, and TOO.

**Table 2:** Surgical interventions of total cohort and subgroups of individuals presenting with hEDS, lacking hEDS or other CTDs (Unaffected), lacking hEDS but with any additional CTD (Any CTD; EDS neg.), presenting with CMI, and presenting with both hEDS and CMI diagnoses (EDS and CMI).

Surgery	Total cohort	hEDS	Unaffected	Any CTD (EDS neg.)	CMI	EDS and CMI
Individuals, *n* (%)	612	404	203	5	396	236
Craniocervical fusion	373 (60.9%)	293 (72.5%)	76 (37.4%)	4 (80.0%)	224 (56.6%)	170 (72.0%)
Posterior fossa decompression	314 (51.3%)	192 (47.5%)	119 (58.6%)	3 (60.0%)	284 (71.7%)	166 (70.3%)
Tethered cord release	245 (40.0%)	189 (46.8%)	55 (27.1%)	1 (20.0%)	134 (33.8%)	105 (44.5%)
Ventriculoperitoneal shunt	82 (13.4%)	56 (13.9%)	25 (12.3%)	1 (20.0%)	65 (16.4%)	42 (17.8%)
Styloidectomy	61 (10.0%)	51 (12.6%)	9 (4.4%)	1 (20.0%)	36 (9.1%)	33 (14.0%)
CSF leak repair	59 (9.6%)	47 (11.6%)	11 (5.4%)	1 (20.0%)	38 (9.6%)	30 (12.7%)
Anterior cervical discectomy and fusion	44 (7.2%)	29 (7.2%)	15 (7.4%)	0 (0.0%)	34 (8.6%)	20 (8.5%)
Transoral odontoidectomy	12 (2.0%)	5 (1.2%)	7 (3.4%)	0 (0.0%)	11 (2.8%)	5 (2.1%)

Percentage is based on the individual subgroup population, *n*.

**Figure 4:**
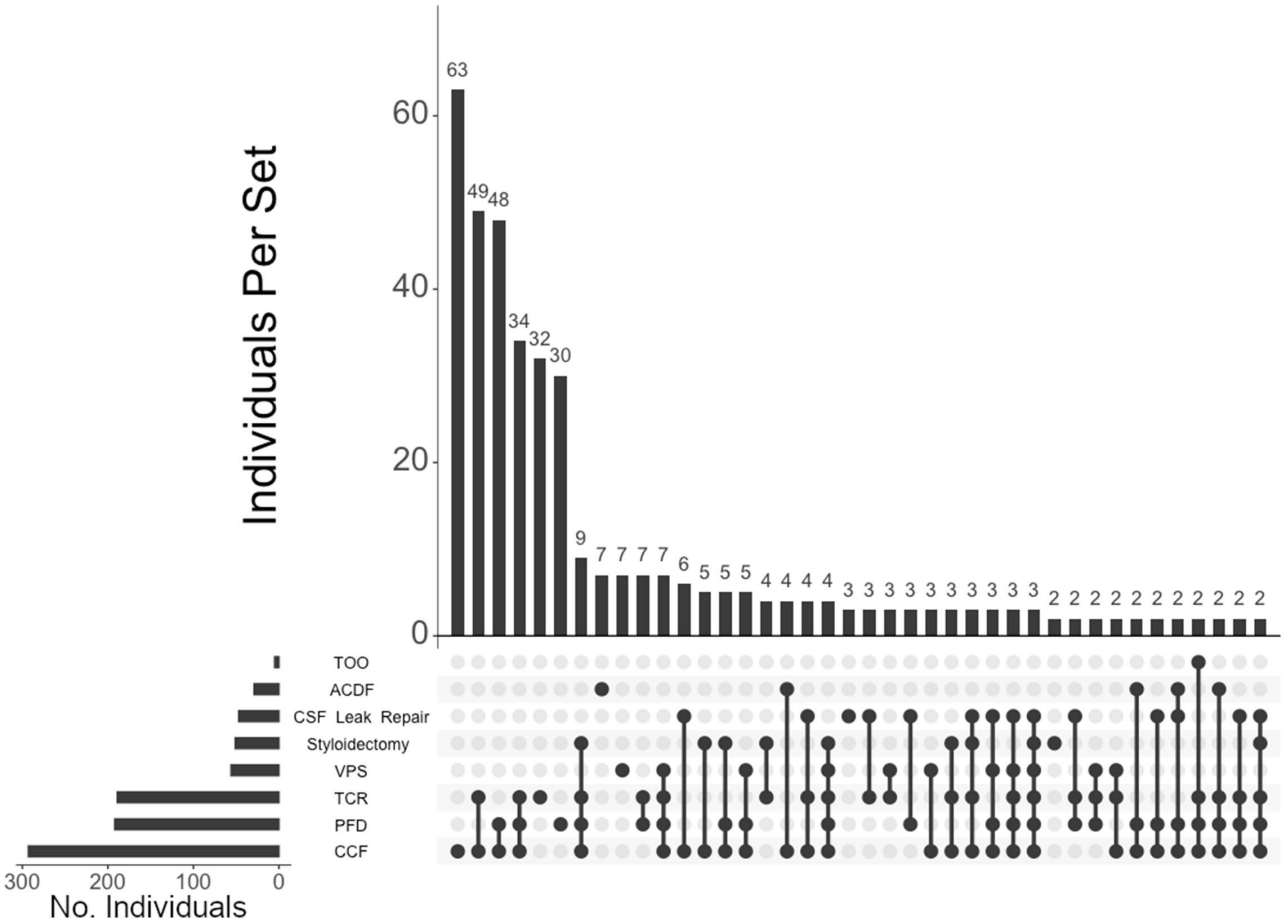
**UpSet plot of the most frequent surgical intersections among those presenting hEDS**. Each column represents the intersection (or lack thereof) of one or more surgeries, with the number of individuals in each grouping listed on top of each bar. Each row indicates groupings of data (sets) corresponding to the surgeries listed on the left. Dark circles indicate the placement of a surgical procedure within a group. Groupings comprised of two or more procedures are indicated by circles connected by a line. Data corresponds to the frequency detailed in Table 2. The top 40 intersections are illustrated. Surgical procedures: craniocervical fusion (CCF); posterior fossa decompression (PFD); tethered cord release (TCR); ventriculoperitoneal shunt (VPS); styloidectomy; CSF leak repair; anterior cervical discectomy and fusion (ACDF).

**Figure 5:**
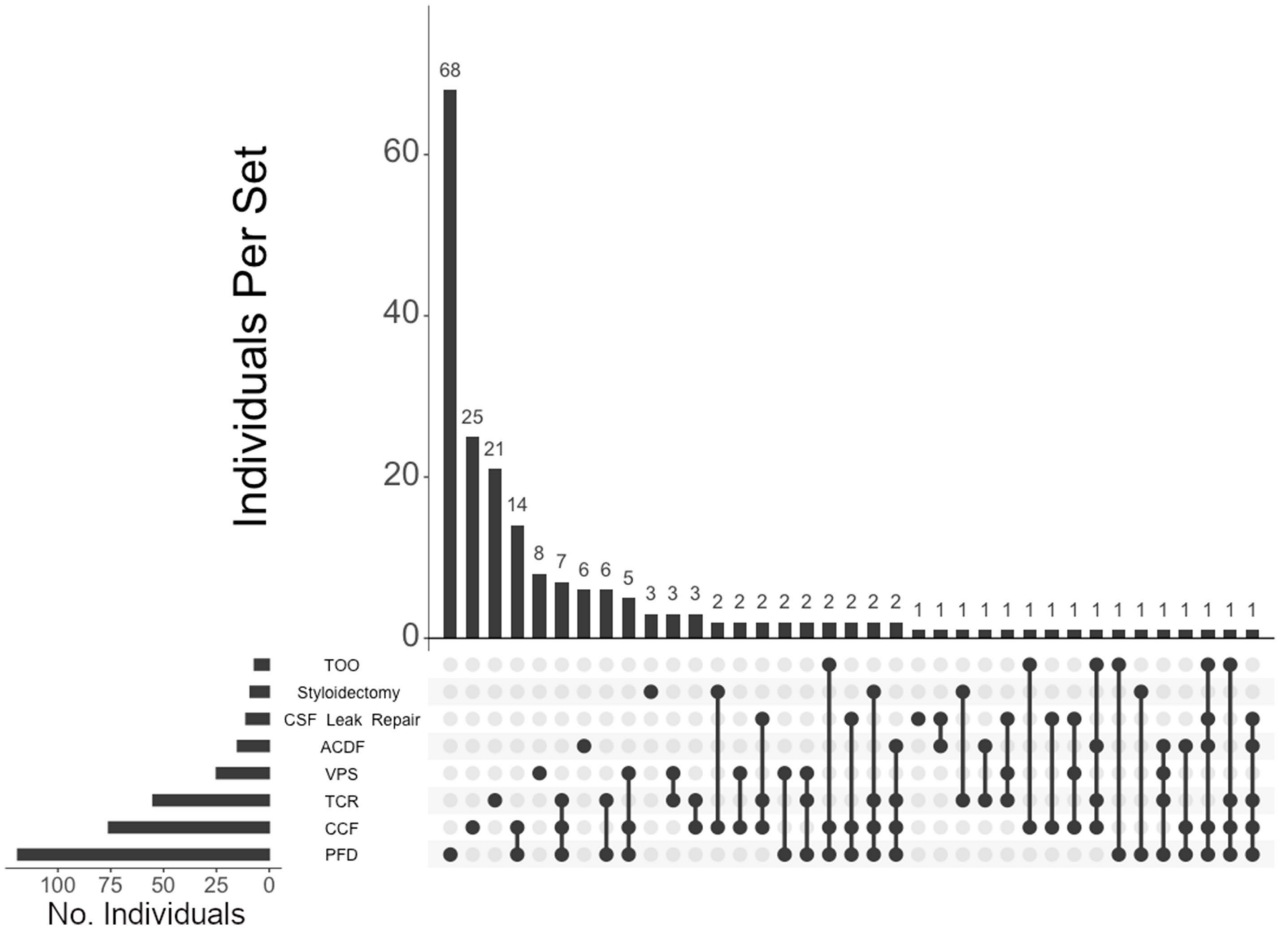
**UpSet plot of the most frequent surgical intersections among the individuals unaffected by CTDs**. Each column represents the intersection (or lack thereof) of one or more surgeries, with the number of individuals in each grouping listed on top of each bar. Each row indicates groupings of data (sets) corresponding to the surgeries listed on the left. Dark circles indicate the placement of a surgical procedure within a group. Groupings comprised of two or more procedures are indicated by circles connected by a line. Data corresponds to the frequency detailed in Table 2. The top 40 intersections are illustrated. Surgical procedures: posterior fossa decompression (PFD); craniocervical fusion (CCF); tethered cord release (TCR); ventriculoperitoneal shunt (VPS); CSF leak repair; styloidectomy; anterior cervical discectomy and fusion (ACDF); transoral odontoidectomy (TOO).

**Figure 6:**
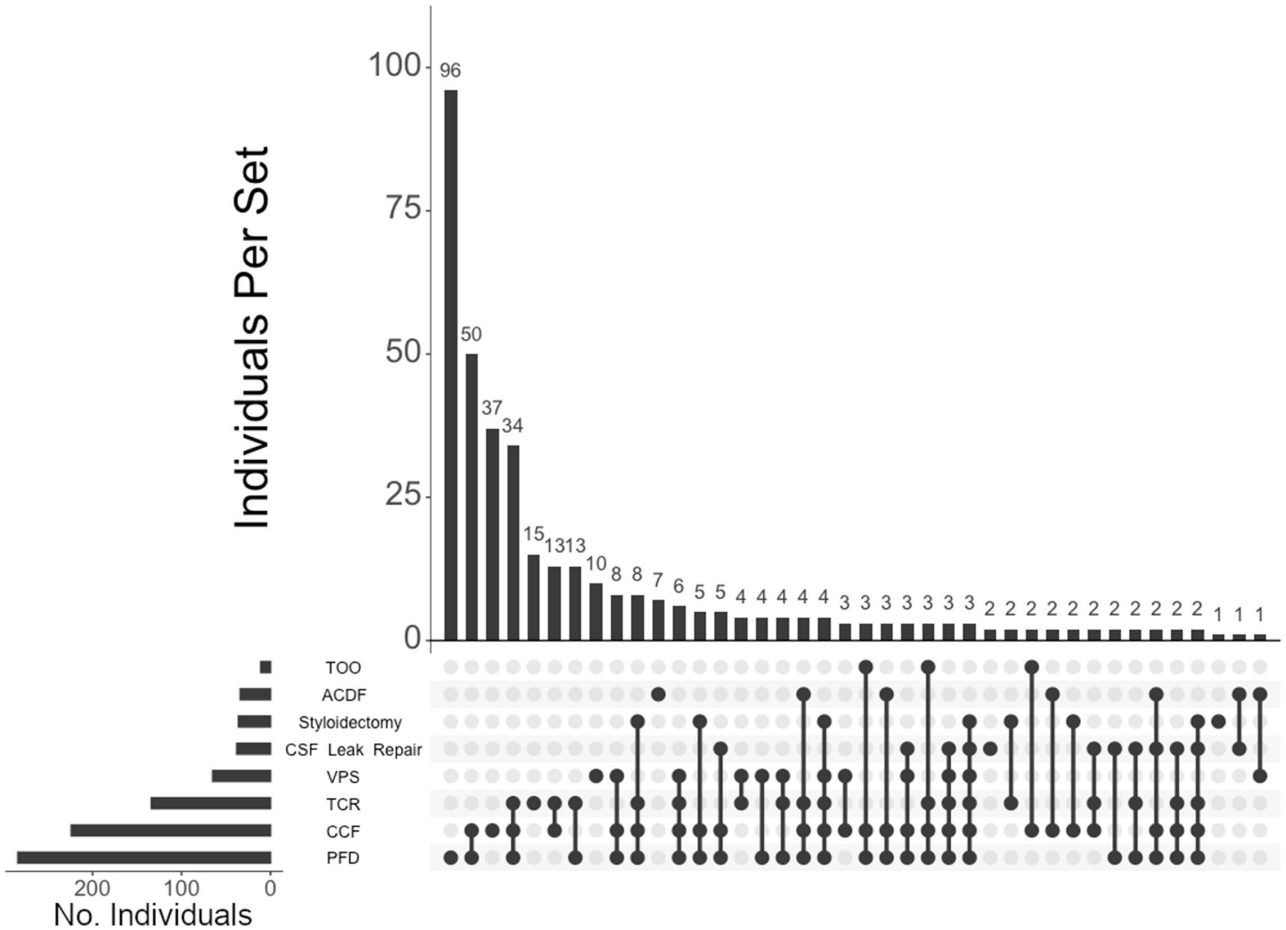
**UpSet plot of the most frequent surgical intersections among individuals presenting with CMI**. Each column represents the intersection (or lack thereof) of one or more surgeries, with the number of individuals in each grouping listed on top of each bar. Each row indicates groupings of data (sets) corresponding to the surgeries listed on the left. Dark circles indicate the placement of a surgical procedure within a group. Groupings comprised of two or more procedures are indicated by circles connected by a line. Data corresponds to the frequency detailed in Table 2. The top 40 intersections are illustrated. Surgical procedures: posterior fossa decompression (PFD); craniocervical fusion (CCF); tethered cord release (TCR); ventriculoperitoneal shunt (VPS); CSF leak repair; styloidectomy; anterior cervical discectomy and fusion (ACDF); transoral odontoidectomy (TOO).

**Figure 7:**
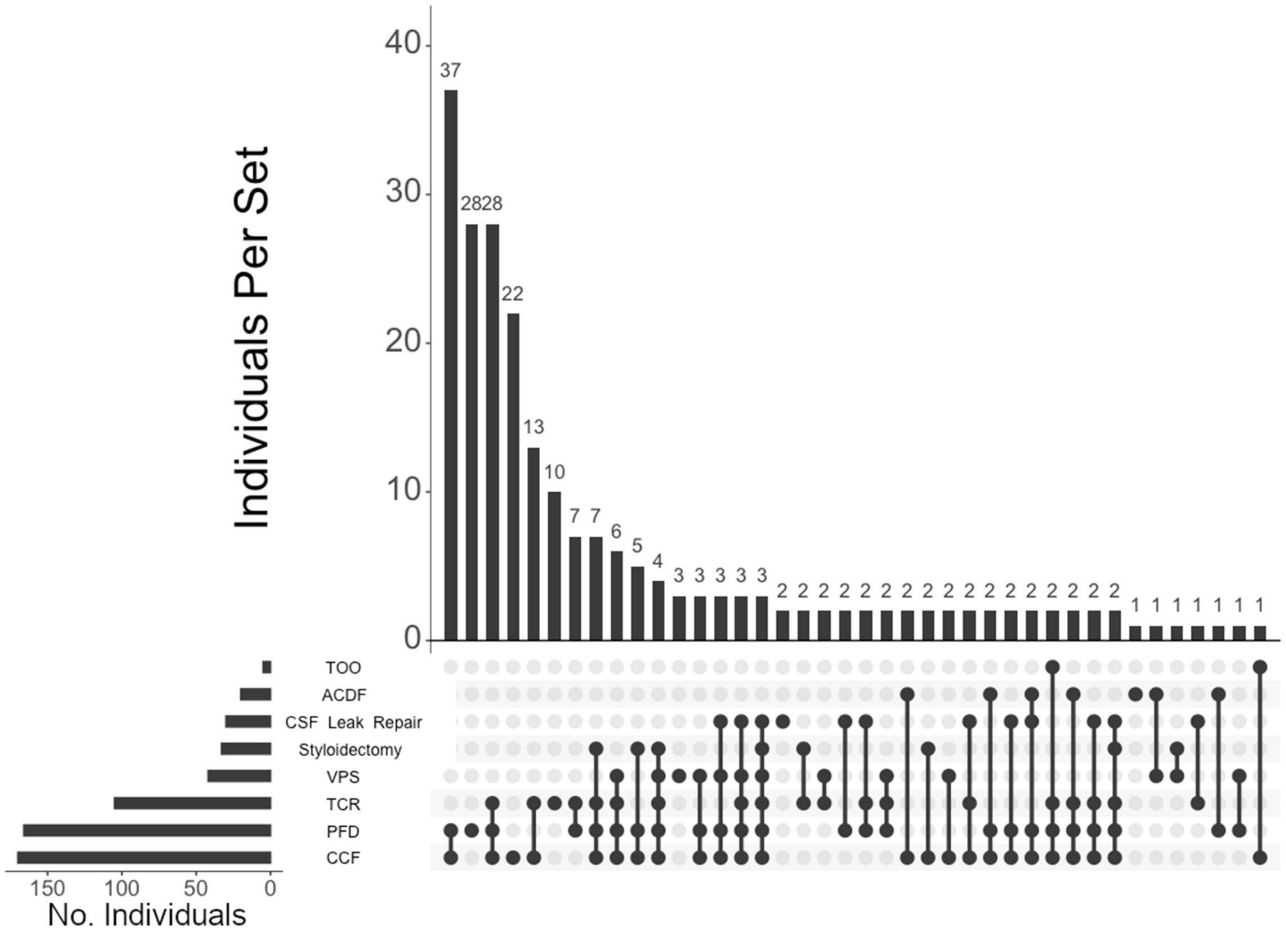
**UpSet plot of the most frequent surgical intersections among individuals presenting with both hEDS and CMI diagnoses**. Each column represents the intersection (or lack thereof) of one or more surgeries, with the number of individuals in each grouping listed on top of each bar. Each row indicates groupings of data (sets) corresponding to the surgeries listed on the left. Dark circles indicate the placement of a surgical procedure within a group. Groupings comprised of two or more procedures are indicated by circles connected by a line. Data corresponds to the frequency detailed in Table 2. The top 40 intersections are illustrated. Surgical procedures: craniocervical fusion (CCF); posterior fossa decompression (PFD); tethered cord release (TCR); ventriculoperitoneal shunt (VPS); styloidectomy; CSF leak repair; anterior cervical discectomy and fusion (ACDF); transoral odontoidectomy (TOO).

Of the 460 individuals diagnosed with hEDS, 404 (88%) chose surgical intervention; 73% of individuals with hEDS required CCF for craniocervical instability and 48% sought PFD (Table 2). Comparatively, 203 of the 250 (81%) of unaffected individuals chose surgical intervention, with PFD being the most frequent (59%) followed by CCF (37%). In the 396 individuals with CMI who underwent surgery, 57% had CCF and 72% underwent PFD. For the 236 individuals diagnosed with both hEDS and CMI, the frequency of both CCF and PFD surgeries were nearly equal at 72 and 70%, respectively. In our hEDS cohort, the most frequent surgeries were (1) CCF alone (63, or 16%), followed by (2) those requiring CCF and TCR (49, or 12%), (3) CCF and PFD (48, or 12%), (4) CCF, PFD, and TCR (34, or 8%), (5) TCR alone (32, or 8%), and (6) PFD alone (30, or 7%) (Figure 4). For unaffected individuals, the most frequent intersections of surgical interventions were (1) PFD alone (68, or 34%), (2) CCF alone (25, or 12%), (3) TCR alone (21, or 10%), (4) PFD and CCF (14, or 7%), and (5) VPS (8, or 4%) (Figure 5) For individuals with CMI, the most frequent intersections of surgical interventions were (1) PFD alone (96, or 24% of those with CMI diagnosis), (2) PFD and CCF (50, or 13%), (3) CCF alone (37, or 9%), and (4) PFD, CCF, and TCR (34, or 9%) (Figure 6). For individuals presenting with both hEDS and CMI diagnoses, the most frequent intersections of surgical interventions were (1) both CCF and PFD (37, or 16%), (2) PFD only (28, or 12%), (3) CCF, PFD, and TCR (28, or 12%), and (4) CCF alone (22, or 9%) (Figure 7).

**Figure 8:**
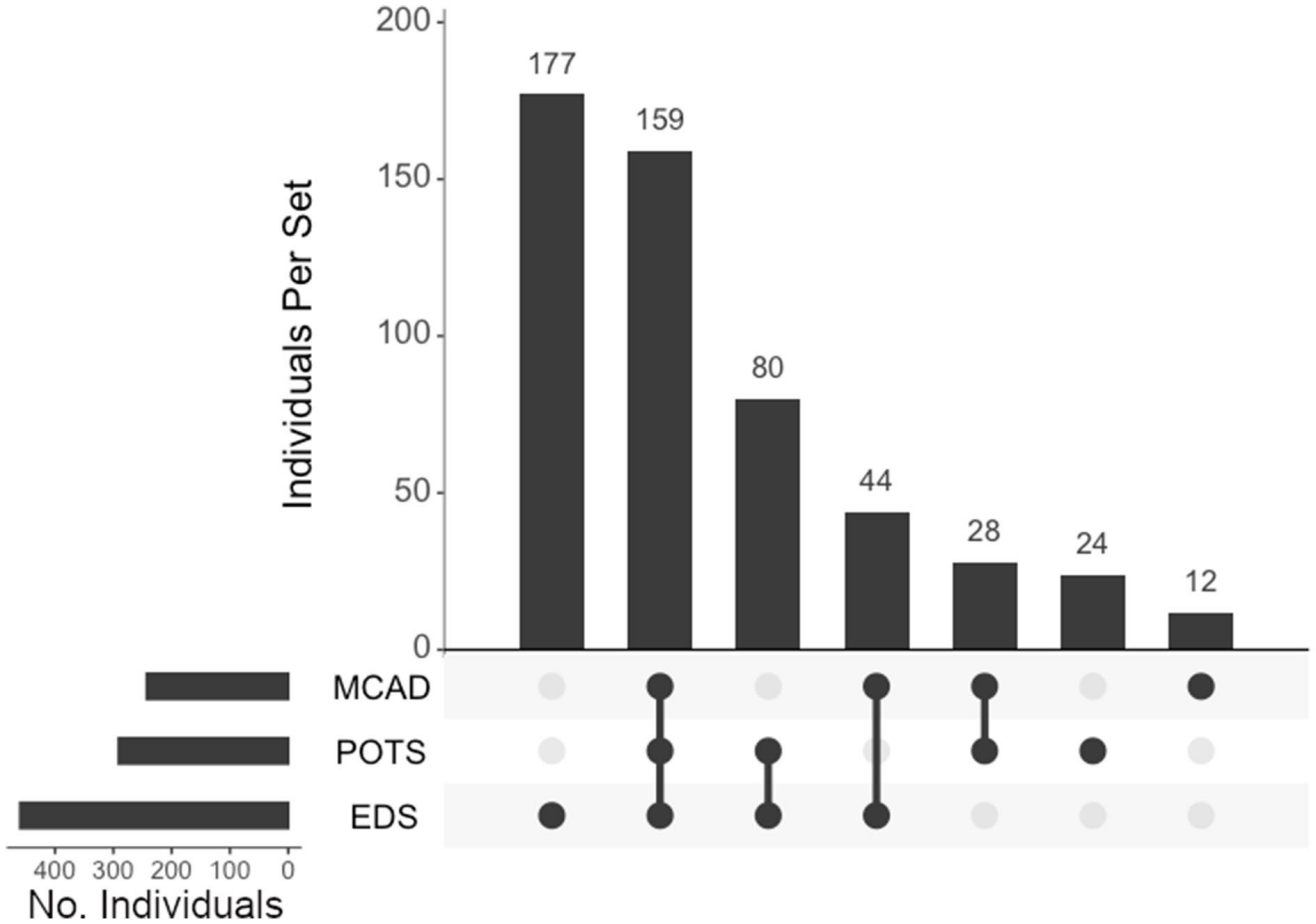
**UpSet plot of the intersections of individuals with hypermobile EDS (EDS), postural orthostatic tachycardia syndrome (POTS), and/or mast cell activation Disorder (MCAD)**. Each column represents the intersection (or lack thereof) of one or more diagnoses, with the number of individuals in each grouping listed on top of each bar. Each row indicates groupings of data (sets) corresponding to the diagnosis listed on the left. Dark circles indicate the placement of a diagnosis within a group. Groupings comprised of two or more comorbidities are indicated by circles connected by a line. Total individuals, *n* = 524.

We assessed individuals who presented with either hEDS, POTS, and/or MCAD and their diagnostic overlaps (Figure 8). Of the 524 who presented with any of these diagnoses, 177 (34%) presented with only hEDS, 159 (30%) had all three diagnoses, 80 (15%) had EDS and POTS, 44 (8%) had EDS and MCAD, 28 (5%) had POTS and MCAD, 24 (5%) presented with POTS alone, and 12 (2%) presented with MCAD alone.

### Associations of diagnoses and surgeries

We isolated data with respect to all diagnoses and surgeries to understand patterns of comorbid interactions, as illustrated in Supplementary Table S2. Multivariate analysis of all diagnoses and surgeries among the total cohort indicated significant patterns associated between multiple sets (Supplementary Tables S3, S4) and is discussed in detail, below.

## Discussion

This was an eight-year retrospective study on individuals referred to our surgical center for suspected neurosurgical problems secondary to diagnosed or suspected CTDs. Previous studies have suggested that neurosurgical comorbidities should be considered in individuals with CTDs (8, 12, 16, 18, 34–38); however, our study is the first to evaluate neurosurgical comorbidities and interventions in a large cohort of these individuals. Herein, we found that most of the individuals in our cohort present with hEDS or CMI (89%, alone or in combination) with a host of associated comorbid musculoskeletal, immunological, and autonomic deficiencies. Further, we found that individuals with CTDs, particularly hEDS, have a distinct grouping of comorbidities and that these comorbidities required neurosurgical intervention in the majority of cases.

Though CMI is defined as a distinct neuroanatomic pathology, it has been suggested that CMI and hEDS are coincident pathologies (17, 39). The findings in our cohort support this observation with over half of both hEDS and CMI diagnoses presenting for its counterpart. Further, we found that individuals with hEDS have additional neurosurgical considerations, requiring intervention for TCS and SH. Nearly half of the individuals in our cohort with hEDS were diagnosed with a tethered cord (49%) and nearly one-quarter were diagnosed with SH (24%). Additionally, individuals with either TCS or SH reported a high rate of hEDS comorbidity (75 and 76%, respectively).

Consistent with previous reports (8, 10, 19, 39, 40), the demographics for this population skews heavily female (over 6:1), and is appreciatively younger than the expected population requiring neurosurgical intervention for severe and progressive musculoskeletal disability (37).

Given the bias toward a female population, along with notable increases in gynecological issues often requiring surgical intervention (40–42), hypermobility-related neurophysiological dysfunction and its downstream impact may be an important consideration in women’s health.

Hypermobility has been shown to be related to the abnormal mechanics of the craniocervical junction (CCJ) (37, 43). Recent imaging studies strongly indicate that the laxity in ligaments comprising the myodural bridges and other suspension ligaments are involved in the pathogenesis of craniocervical instability (CCI) (35). Given the inherent connective tissue laxity in the hEDS population, the risk of CCI is assumed to be a substantially higher (44). This is consistent with our observations that the majority of the individuals in our cohort with hEDS presented with severe structural compromise of the CCJ, requiring neurosurgical intervention. Among those with hEDS, 64% required CCF to treat CCI, and among all who underwent CCF surgery, 79% presented with an hEDS diagnosis. Additionally, individuals with hEDS in our cohort had unique neurophysiological considerations aside from CCI that necessitated neurosurgical interventions beyond CCJ stabilization. For example, a diagnosis of hEDS was found in the majority of individuals undergoing styloidectomy (84%), CSF leak repair (80%), TCR (77%), VPS (68%), ACDF (66%), and PFD (61%). Interestingly, despite their co-incidence, hEDS and CMI surgical profiles are seemingly distinct. Our study shows that hEDS significantly correlates with CCF (*p* < 0.001), TCR (*p* < 0.001), styloidectomy (*p* < 0.001), and CSF leak repair (*p* < 0.01) surgeries while CMI correlates with PFD (*p* < 0.001), VPS (*p* < 0.001), ACDF (*p* < 0.05), and TOO (*p* < 0.05). Thus, while these populations overlap, they require differing surgical interventions to address comorbidities specific to each subtype. A direct comparison of rates of diagnoses and surgeries between the hEDS and unaffected subgroups showed no statistical significance in PTLDS presentation. Further, there was no association of the unaffected individuals with PFD, VPS, ACDF, and TOO surgeries. In contrast, we did find that individuals with hEDS were more likely to present with all diagnoses other than CMI and MALS as compared to the unaffected group.

In addition to neurosurgical considerations displayed among our population, we found significant associations between immune- and autonomic-related comorbidities and hEDS. Anecdotal reports have suggested that individuals with hEDS often present with concomitant POTS and MCAD, which may have overlapping symptoms (10, 15, 45). We believe this report is the first large population study to confirm this finding, define this subgroup, and support the association of this hEDS/POTS/MCAD diagnostic triad. In our cohort, 52 and 44% of individuals presenting with hEDS were co-diagnosed with POTS and MCAD, respectively. Of the individuals presenting with POTS and MCAD in our cohort, 82 and 84%, respectively, were coincident with hEDS. In the total cohort, 524 (73%) presented with at least one diagnosis with this triad. Diagnosis intersections indicated that, of the 460 individuals presenting with hEDS, the largest comorbid association was with both POTS and MCAD, with 159 individuals (35% of the hEDS cohort) exhibiting all three diagnoses, followed by 80 individuals with concurrent hEDS and POTS, and 44 individuals with both hEDS and MCAD. Surgically, the populations of all three disorders correlate significantly with CCF (*p* < 0.001), TCR (*p* < 0.001), and CSF leak repair (hEDS, *p* < 0.01; POTS, *p* < 0.05, and MCAD, *p* < 0.001), indicating clear overlaps in phenotype displayed among individuals in this hypermobility triad.

As noted above, we found that CMI is strongly associated with the population unaffected by CTDs; however, while this subgroup required surgical intervention for their CMI, these individuals had considerably lower rates for the autonomic and immunological dysfunction as compared to those with hEDS. Rates for POTS, MCAD, and dysautonomia in hEDS patients are 2.5- to 3.8-times the rate in the unaffected despite CMI and PFD rates being more similar. For this reason, as noted above, we cannot exclude the possibility that CMI in our so-called unaffected group may be related to other undiagnosed or sub-clinical mesenchymal disorders distinct from hEDS, which is consistent with the fact that these individuals were referred to us with a suspicion of CTD.

We also found multiple other associations with hEDS and CMI. There is a heavy enrichment of hEDS comorbidity in individuals also diagnosed with gastroparesis (89%), MALS (88%), dysautonomia (87%), SFPN (84%), GERD (79%), hypothyroidism (74%), ME/CFS (72%), and PTLDS (70%). Notably, individuals with hEDS bear these comorbidities far more than those exhibiting CMI in our cohort. In a direct comparison of individuals with hEDS and those with CMI, we found that POTS, MCAD, dysautonomia, and ME/CFS rates were approximately 1.5 to 2-times higher in hEDS than CMI.

Overall, we found that there are multiple significant associations among diagnoses and required neurosurgical interventions within subgroups of the total cohort. Notably, the majority of this population does not fit cleanly into one diagnostic category, but rather separates into clusters of comorbidities that likely represent different syndromes and require distinct management. Future onboarding procedures may do well to incorporate diagnostics for all associated comorbidities in patient intake and evaluation criteria.

### Limitations

Notably, the diagnostic criteria for hEDS and related disorders has evolved over time. As recently as 2017, a new consensus was reached for revising the International hEDS Classification (11), well within the time frame of those reviewed here. Of note, where fatigue is mentioned in the revised hEDS classification, ME/CFS—a condition now recognized to be frequently associated with hEDS (46, 47)—is not specifically addressed. As the changing clinical criteria “moves the goal posts” when assigning proper diagnoses, older data may not be as robust when compared to more current intakes. Within our own intake forms, questions related to relevant personal history have been added as newer comorbidities linking to hEDS became apparent (e.g., styloid hypertrophy, ME/CFS). For this reason, we believe some of these concomitant disorders may be underdiagnosed and/or underreported.

The individuals examined for this report constitute the severe end of the functional spectrum, so findings presented herein may not be generalizable to all individuals within the hEDS population. Further, as a designated center for neurosurgical intervention, we are more likely to encounter such a severely afflicted population. However, we believe general awareness of the unique neurosurgical considerations of this population is important should individuals with hEDS diagnoses develop any of these issues. On-boarding and health survey questions pertaining to past health (e.g., diagnosis predating our interaction, accurate surgical and clinical history, etc.) are taken in cooperation with the individual and supporting documentation from other medical facilities is not always provided.

## Conclusion

We present a retrospective study of diagnoses, comorbidities, and neurosurgical interventions on the largest cohort of individuals presenting CTD-related neuromuscular dysfunction to date. Herein, this study revealed hEDS to be the most common CTD of our population, which we found significantly associates with unique musculoskeletal comorbidities requiring skilled and experienced neurosurgical intervention and management. Further, this neurophysiological dysfunction presents itself with a syndrome of coincident autonomic and immune disorders, fostering additional data that provide clear and observable signs to both monitor and anticipate prognosis for this complex population. Increased awareness of these concurrent pathologies should afford early identification and subsequent referrals to qualified centers with experience in CTD-based neurophysiological treatment. Critically, rigorous management and follow-up studies may provide the means for clarifying – perhaps redefining – the diagnostic markers of extreme hypermobility pathologies.

## Data Availability

The original contributions presented in the study are included in the article/Supplementary material, further inquiries can be directed to the corresponding author.

## Supplementary material

SUPPLEMENTARY TABLE S1 Chart review data for the total cohort (sex, age, diagnoses, and surgeries). Positive indication for a diagnosis or undergoing surgery indicated by 1. Totals of each set, in number (*n*) and percentage of the full cohort (%), are indicated at the bottom.

SUPPLEMENTARY TABLE S2 Tabular representation of diagnosis (orange) and surgery (pink) within each diagnostic and surgical cohort. Each cohort, by row, indicates the number of individuals in that set (*n*, grey) with matrix numbers corresponding to percent of individuals with diagnoses and surgeries corresponding to that set.

SUPPLEMENTARY TABLE S3 Heatmap of statistical correlations among all diagnosis and surgical interventions within the total cohort. Levels of significance are color-coded for each subcategory interaction [**p* < 0.05 (red), ***p* < 0.01 (yellow), and ****p* < 0.001 (green)]. A comprehensive accounting of these relationships is detailed in Supplementary Table S4

SUPPLEMENTARY TABLE S4 Statistically significant correlations among diagnoses and surgeries. Data mirrors the graphic matrix depicted in Supplementary Table S3.

## Glossary

ACDF: Anterior Cervical Discectomy and Fusion
CCF: Craniocervical Fusion
CCI: Craniocervical Instability
CCJ: Craniocervical Junction
CMI: Chiari I Malformation
CMS: Cervical Medullary Syndrome
CTD: Connective Tissue Disorder
EDS: Ehlers Danlos Syndrome
hEDS: Hypermobile Ehlers Danlos Syndrome
GERD: Gastroesophageal Reflux Disease
MALS: Median Arcuate Ligament Syndrome
MCAD: Mast Cell Activation Disorder
ME/CFS: Myalgic Encephalomyelitis/Chronic Fatigue Syndrome
PFD: Posterior Fossa Decompression
POTS: Postural Orthostatic Tachycardia Syndrome
PTLDS: Post-Treatment Lyme Disease Syndrome
SFPN: Small Fiber Polyneuropathy
SH: Styloid Hypertrophy
TCR: Tethered Cord Release
TCS: Tethered Cord Syndrome
TOO: Transoral Odontoidectomy
VPS: Ventriculoperitoneal Shunt
